# Dental Education

**Published:** 1871-06

**Authors:** 


### DENTAL REGISTER.

VOL. XXV.] JUNE, 1871. [No. 6.

### Communications.

### DENTAL EDUCATION.

Read before the Michigan State Dental Society.

Much has been written, and to the point too, on this subject. Much has been done in the education of dentists; so much that even at this early day in the history of our specialty in the art of cure, dentistry is almost recognized as a learned profession. But when we look at the masses of so-called dental practitioners, when we examine dental literature, and when we question ourselves as to our theoretical as well as practical attainments, we find there is very much yet to be accomplished in this direction. If dentistry were a mechanical trade, as too many consider it, even then a certain amount of scientific knowledge would be of much importance to the operator. But if it be a profession—a specialty of cure, or of the healing art, or of medicine, as you may see fit to call it; if the dentist be a curist, a conservator of the health of mankind, and a healer of the diseases of his neighbors, then how pre-eminently important is it that he should be acquainted with every science, that he should question every source of information and every avenue of knowledge that can assist him in the performance of his most responsible and humane duties.

In order to prepare ourselves for the proper consideration of this subject—Dental Education—let us spend a few moments in considering the question, what is dentistry?

In the earlier history of dentistry as a separate business, the man who, with strong arm and senseless brain, could mutilate his fellow by tearing out his teeth, and then supply the place of those teeth with some sort of an artificial substitute, was called a dentist. Then the treatment of some of the simpler diseases of the teeth was added to his occupation. Still the extraction of teeth and the manufacture of artificial dentures occupied his principal attention—so much so, that a student, or more properly an apprentice, taken into an office to learn dentistry, was considered sufficiently qualified to set up business on his own account, so soon as he could make artificial teeth, and mount them on a plate suitable to be worn by his patron, and perhaps, put fillings in a few simple cavities that would stay long enough for him to collect pay for the operations, and frequently he was not required to learn to make artificial teeth—for this branch of the business was soon, very wisely, adopted as a specialty by men of ability and untiring industry, who devoted their whole time and energies to this manufacture, supplying an article almost infinitely superior to any teeth that would have been produced in any other way. Thus the mounting and inserting of artificial teeth has been handed down from tutor to learner as a most important part of the business of the dentist. (One principal reason why this system has been adopted is, that it is pecuniarily advantageous, for the student can soon learn to earn something in the laboratory—while his pursuit of scientific knowledge adds nothing to his tutor's income.) If proof were required to substantiate these statements, it would only be necessary to refer to the transactions of dental societies. We would then find that not more than ten or fifteen years ago the time of the sessions of the dental societies then in existence, was largely, and in many cases principally occupied in the discussion of subjects pertaining to what was called "Mechanical Dentistry," and the dentist went home and talked artificial teeth so long, so much, and so persistently, frequently going around from house to house peddling his wares, that the public soon learned to consider a dentist a man skilled in "pulling teeth" and in making artificial substitutes, and to look upon dentistry as a mechanical trade, and the "sober second thought of the people," or public opinion, which is seldom wrong, was certainly not wrong in this case. For dentistry as then practiced, and as now carried on by many so-called dentists, is simply a mechanical trade—a manufacture.

But is this manufacture of artificial dentures, dentistry? Looking from our present advanced standpoint, I say no. To illustrate: The physician who devotes attention to the cure of cutaneous diseases, finding his best skill baffled by a severe case of scald head, may devote a part of his time to the manufacture of an artificial substitute for the natural capillaries he could not save. But, while he is at work at wig making, is he practicing medicine? The general surgeon may occupy a part of his time in making artificial limbs to supply the place of those he has been obliged to remove to save his patients lives. But is he practicing surgery while carving out wooden legs? or is the artificial limb maker a surgeon? Just so the dentist may spend a part of his time making artificial dentures, but while so engaged he is not practicing dentistry. He is engaged in a business outside of his profession. He is simply a manufacturer. And the sooner this manufacture is entirely divorced from dentistry, the better for the dentist, and the better for his patient.

Let the artificial teeth mounter follow the worthy example set him by the artificial teeth maker, and devote his whole time and attention to his branch of manufactures Don't give him the forceps, nor the turnkey, nor even the hammer and nail set, not anything with which he can extract teeth, any more than you would give the surgeon's knife and saw to the artificial limb maker. Give all operations on the natural organs of the oral cavity to the dentist, and when his skill fails to save the teeth, let *him* remove the diseased roots, and then turn the case over to the manufacturer of artificial dentures. Then the dentist, untrammeled by a mechanical trade as a part of his business, may take his proper position in the family of curists.

We are now prepared to answer the question—What is dentistry? I answer, as before intimated: Dentistry is a specialty of cure, taking cognizance of the diseases of the oral cavity. It includes both medicine and surgery. The dentist is a person who, being educated therefor, devotes his time and talents, his abilities and acquirements, and his skill, to the prevention and cure of the diseases of the organs of mastication. Dentistry is a most important specialty. For, if the very gateway to *nutrition* be obstructed, this process is impaired, and without healthy nutrition, the whole body must suffer; nor is dentistry any the less honorable or responsible for being called a specialty. Medicine and surgery are but specialties of the healing art. The intelligent and honorable medical practitioner will, when applied to for the cure of certain diseases, send his patient to the surgeon or the dentist. The general surgeon also frequently has occasion to send a patient to the physician or the dentist, and the medical man, who practices surgery as well, must sometimes call in the aid of the dentist. So the dentist may ask the counsel, or turn his patient over to the care of the physician or general surgeon, when the disease he is called upon to treat, is in part or wholly outside of his specialty. Mutual relationship and dependence must exist between the different members of the healing profession, and when dentists cease to make a mechanical trade their principal business, and learn and practice their profession as a healing art, then will that relationship be *cheerfully acknowledged*, and, in my opinion, not till then. So that, notwithstanding the physician has a larger range of diseases to treat than the dentist, and his practice is more general, it is not universal, and therefore is special. And if medical practice were subdivided into more specialties, it would be a blessing to suffering humanity, and medicine would become progressive as dentistry now is.

For life is too short, and the mind of man too weak to allow any one person to become thoroughly acquainted with the pathology and cure of all the diseases that afflict poor human nature. If one class of physicians would devote special attention to the study and cure of diseases of the lungs and thoracic viscera, and another to the bowels and genital organs, another to the brain and nervous system, another to the eye and ear, as an another class now does to the organs of mastication, the afflicted could apply to the doctor with much greater certainty of a speedy and permanent cure than he now does. And here I wish to say, by the way, that I consider the action of the American Medical Society, in prohibiting its members from practicing, or advertising as specialists, other than in the general terms of "physician and surgeon," as retrogressive. It is putting a block before the wheel of progress. It is fighting against the logic of events. And I hope to see the time when the live, progressive men of that society will demand its reconsideration.

But to return: We have seen that the dentist is a special physician and surgeon, and we are therefore forced to the necessary conclusion that he who is not possessed of the education and skill requisite to make him a dental physician and surgeon, is not a dentist, and should not be recognized as such by the public. What, then, should be the education of the coming dentist? Evidently, in general terms, such as to prepare him to practice medicine and surgery as a dental specialty. His education should be as thorough and complete as that of the general physician and surgeon. He need not, necessarily, be able to prescribe or operate for the cure of diseases outside of his specialty; but as the general principles and the fundamental sciences that underlie and form the superstructure of the science and art of healing are the same in all departments of cure, they should be equally well understood by all curists. Many of us can look back to the time when the facilities for acquiring a dental education were very poor. They consisted of the privilege of paying a practicing dentist a certain fee for the opportunity to work in his laboratory a few months or years, and occasionally witnessing a few of his operations. We might read his books, if he had any, but he had no time to examine us in our studies, or to assist us in overcoming the obstacles that beset our path, only so far as would enable us to become more profitable apprentices. But the spirit of progress which pervades all departments of science and art, has worked a great change in this respect; and, thanks to the demands of the public—thanks to associated effort—thanks to dental literature—thanks to dental colleges—the facilities now offered the student of dentistry for acquiring a thorough professional education are equal to those furnished by any other profession in this country, and the members of this society are sound on this question, for in 1866 we adopted the following resolution:

"That it be deemed derogatory to the best interests of the profession for any member to take students for a less time than three years, and under no circumstances unless he pledge himself to attend two terms in a dental college."

Also—

"*Resolved*, That any person entering the profession after this date shall not be eligible to membership in this association until he has graduated at some regular dental college."

So that, as far as we are concerned, the coming dentist must be an educated man. But what can we do for those poor deluded mortals, who, upon hearing our terms to pupils, in accordance with the above resolutions, leave our office doors and go to work for our neighbors, and in three months, more or less, hang out their shingle as dentists? Or rather what can we do to save the too credulous public from mutilation and injury by this class of ignoramuses? I can see but one feasible plan to accomplish this desirable object, and that is this: inasmuch as the manufacture of artificial dentures is the principal business of this class of operators, let us give them the whole of it, and let the public understand that the inserting of artificial teeth, except on the natural roots, is not dentistry, and that the artificial denture maker is not a dentist, and should not be employed to perform dental operations any more than the artificial limb maker should to perform operations in general surgery. And let him who proposes to practice dentistry prepare himself by making use of the best educational facilities offered to practice his profession with skill and fidelity to the weal of his patients; and I would go farther; I would by legal enactment prohibit any one from commencing the practice of any branch, system or specialty of medicine or surgery in this State unless he be a graduate of a legally authorized medical or dental college or university. And I would have those now practicing subjected to a reasonable examination and license, and I would prohibit every one from prescribing or administering a dose of medicine to or performing any surgical operation on a human being, for pay or reward, without such diploma or license. I would thus do all that education and the law can do to save the people of this State from the evils of charlatanry and malpractice; and further, to carry out these ideas, I would have the chair of "Mechanical Dentistry" taken out of the faculties of our dental colleges, drop the teachings of a mere mechanical business, the manufacture of artificial dentures from their curiculum, and devote the time thus gained to teaching how to save the natural organs, make dentistry a strictly conservative profession. Not that the mechanical business of manufacturing artificial dentures is not honorable, for it is just as honorable and just as necessary as any other business, and more lucrative than dentistry, but it does not belong with dentistry any more than wig making belongs to medicine. And now let us consider more particularly the dental educational wants of our own State, and propose a plan to supply them. We know there are a decade of good dental schools and university professorships in this country, probably amply sufficient to supply the demands on them. But there is a law in domestic economy that holds good in this case, viz: an increased supply of a good thing generally produces an increased demand. For a larger number of people become acquainted with it, and to know a really meritorious article is to want it. Thus there is a larger proportion of educated dentists in the immediate vicinity of dental colleges than at a great distance from them. Hence if we supply the means of a good and thorough dental education within the doors of our own honored university, the people will not be slow to appreciate and use those facilities; and they naturally look to the Michigan Dental Society as the source whence shall proceed those recommendations and improvements which shall guarantee to the afflicted relief from the various diseases that affect the organs of mastication. In obedience to this demand I would propose to have established a medical or *cure university* for the education of all persons desiring to learn any department, specialty or system of cure. I would admit all who possess the requisite character and preliminary acquirements, and teach them all together in one class or classes, in anatomy, histology, physiology, chemistry, botany, mineralogy, pathology, semeiology, and any other sciences or departments of knowledge that may be common to all curists. Then let each one select his specialty and go to that department and receive a thorough, theoretical and practical education in that specialty. Such an arrangement, faithfully carried out, would produce the best dental school in the world, as well as a first class medical university.

